# Declining trends but persistent disparities in cervical cancer mortality in the United States, 1999–2023

**DOI:** 10.3389/fpubh.2026.1892243

**Published:** 2026-09-03

**Authors:** Jie Liu, Yonglin Liu, Yanjiao Jiang, Xuefeng Wang, Xianwei Zhou

**Affiliations:** 1 Department of Reproductive Medicine, The Second Affiliated Hospital of Zhejiang Chinese Medical University, Hangzhou, China; 2 Department of Gynecology and Obstetrics, Zhujiang Hospital, Southern Medical University, Guangzhou, China; 3 Department of Gynecology and Obstetrics, The Second Affiliated Hospital of Zhejiang Chinese Medical University, Hangzhou, China

**Keywords:** CDC WONDER, cervical cancer mortality, disparities, HPV, temporal trends

## Abstract

**Background:**

Cervical cancer is largely preventable through effective screening and human papillomavirus (HPV) vaccination; however, mortality disparities remain a persistent public health challenge in the United States. This study aimed to examine long-term trends and socio-demographic and geographic inequalities in cervical cancer mortality.

**Methods:**

We conducted a population-based study of cervical cancer mortality among U.S. women aged ≥25 years from 1999 to 2023 using national mortality data from the CDC WONDER database. Age-adjusted mortality rates (AAMRs) were calculated using the 2000 U.S. standard population. Temporal trends were evaluated using Joinpoint regression to estimate annual percent changes (APCs) and average annual percent changes (AAPCs). Analyses were stratified by census region, race/ethnicity, urbanization level, age group, and state. Age restrictions were harmonized across bridged-race and single-race population estimates, and corrected estimates were used for trend analyses.

**Results:**

Overall cervical cancer mortality declined substantially from 1999 to 2023, with the female-specific AAMR decreasing from 4.37 to 2.09 per 100,000 women (AAPC, −3.27%; 95% CI, −3.81 to −2.73). Despite this overall decline, substantial disparities persisted. Mortality rates remained consistently higher in the Southern region and lowest in the Northeast, which showed the greatest reduction over time (AAPC, −4.23%; 95% CI, −5.31 to −3.13). Non-Hispanic Black women experienced persistently higher mortality compared with other racial and ethnic groups, and women residing in nonmetropolitan areas had elevated mortality rates. Considerable geographic variation was observed across states, with mortality rates in higher-burden states approximately two- to four-fold higher than those in lower-burden states. Sensitivity analyses restricted to women aged ≥25 years confirmed consistent long-term mortality trends between bridged-race and corrected single-race estimates. No evidence of an accelerated decline around 2019/2020 was identified, and the previously reported post-2019 acceleration was attributable to inconsistent age restrictions during data extraction rather than a true epidemiological change.

**Conclusion:**

Although cervical cancer mortality has declined significantly in the United States over the past two decades, substantial socio-demographic and geographic disparities persist. These findings highlight the need for targeted public health strategies to improve equitable access to screening, HPV vaccination, and timely treatment, particularly in high-risk populations and underserved regions.

## 1 Introduction

Cervical cancer remains a major cause of cancer-related morbidity and mortality worldwide, despite being largely preventable through effective screening and human papillomavirus (HPV) vaccination. Globally, cervical cancer accounted for an estimated 604,000 new cases and 342,000 deaths in 2020, with a disproportionate burden in low-resource settings (1). For example, in Kenya, cervical cancer is the leading cause of cancer-related death among women, with an estimated 3,200 deaths in 2020 (2). Persistent infection with high-risk HPV is responsible for the vast majority of cervical cancer cases, and the implementation of cytology-based screening and HPV vaccination programs has led to substantial reductions in incidence and mortality in many high-income countries (3, 4).

In the United States, the widespread use of Papanicolaou (Pap) smear screening since the late twentieth century has contributed to a marked decline in cervical cancer incidence and mortality over the past several decades (5). However, recent evidence suggests that this progress has slowed, and in some populations, mortality rates have plateaued or even increased (6).

The risk of cervical cancer mortality is not evenly distributed across populations. Previous studies have demonstrated substantial disparities by race and ethnicity, geographic region, and socioeconomic status (7–9). In particular, NH (non-Hispanic) Black women experience significantly higher cervical cancer mortality than other racial and ethnic groups, and women residing in the southern United States and rural areas bear a disproportionate burden (10). Women with low socioeconomic status are four times more likely to be diagnosed with cervical cancer than those with high socioeconomic status (11). These disparities have been attributed to differences in access to screening, HPV vaccination uptake, healthcare availability, and broader social determinants of health (12).

Although several national surveillance studies have examined temporal trends in cervical cancer mortality in the United States, including recent analyses using CDC WONDER data to characterize cervical cancer trends and demographic patterns (13), previous research has primarily addressed specific aspects of mortality patterns and disparities. However, comprehensive evaluations of mortality disparities across multiple demographic and geographic dimensions, particularly using underlying cause-of-death data and assessing state-level variation over time, remain limited. Understanding these multidimensional disparities is critical for identifying populations at highest risk and informing targeted public health interventions.

Therefore, we aimed to examine long-term trends and disparities in cervical cancer mortality in the United States from 1999 to 2023 using national mortality data. Specifically, we evaluated age-adjusted mortality rates (AAMR) and temporal trends across racial and ethnic groups, geographic regions, individual states, and urban–rural categories, as well as crude mortality rates across age groups.

## 2 Methods

### 2.1 Data source and study design

This study used mortality data from the Multiple Cause of Death (MCOD) database in CDC WONDER, provided by the Centers for Disease Control and Prevention (CDC). The MCOD database contains nationwide death certificate data compiled by the National Center for Health Statistics (NCHS) and provides comprehensive and representative mortality estimates for the U.S. population from 1999 onward. Annual cervical cancer–related deaths and corresponding population estimates were extracted and stratified by sex, race/ethnicity, geographic region, state, and urban–rural classification. Race/ethnicity-specific analyses were restricted to records with known race/ethnicity classification. Deaths with unknown or unclassified race/ethnicity were retained in overall mortality estimates but excluded from race/ethnicity-specific analyses.

Age-adjusted mortality rates (AAMR) were calculated using the 2000 U.S. standard population. Because CDC WONDER transitioned from bridged-race to single-race population estimates during the study period, we evaluated the comparability of mortality estimates derived from these two population frameworks. Bridged-race data were used for 1999–2020, and single-race data were used for 2018–2023. Overlapping years (2018–2020) were assessed to evaluate consistency between the two estimation methods.

A post-hoc audit of CDC WONDER query parameters identified that the initial single-race extraction did not apply the predefined age restriction of ≥25 years, whereas the bridged-race extraction was restricted to women aged ≥25 years. Because cervical cancer mortality was not observed among women younger than 25 years in age-stratified CDC WONDER data, corrected single-race AAMRs for women aged ≥25 years were derived using the corresponding U.S. 2000 standard population weights (274,634,000/177,593,000 = 1.54642). After applying consistent age restrictions, corrected single-race estimates closely matched bridged-race estimates during overlapping years, supporting the comparability of the two data series.

All primary analyses were based on female-specific AAMRs among women aged ≥25 years. The bridged-race series was used for 1999–2020, and corrected single-race estimates were used for 2018–2023 to extend the analysis through the most recent available period.

### 2.2 Case definition

Cervical cancer–related deaths were identified based on the underlying cause of death recorded on death certificates. Cases were defined using the International Classification of Diseases, Tenth Revision (ICD-10) codes C53.0 (malignant neoplasm of endocervix), C53.1 (malignant neoplasm of exocervix), and C53.8 (overlapping lesion of cervix uteri). Deaths were classified as cervical cancer–related only when one of these codes was listed as the underlying cause of death.

### 2.3 Standardization

To account for differences in population age structure, all mortality rates were age-standardized to the 2000 U.S. standard population. The primary measure was the age-adjusted mortality rate (AAMR) per 100,000 population, with corresponding 95% confidence intervals (CI). Because cervical cancer occurs almost exclusively in women, the female-specific AAMR (deaths per 100,000 female population aged ≥25 years, age-standardized to the 2000 U.S. standard population) is the epidemiologically appropriate measure and is used throughout this study.

Because Joinpoint regression does not support age-adjusted analyses for age-specific groups, crude mortality rates were calculated for age-group analyses. Temporal trends were assessed using annual percent change (APC) and average annual percent change (AAPC), with corresponding 95% CIs and *p* values.

### 2.4 Data analysis

During data processing, stratified data files obtained from CDC WONDER were batch imported using R software (version 4.4.2). Key variables, including year, number of deaths, crude mortality rates, age-adjusted mortality rates (AAMR), 95% confidence intervals (CIs), and standard errors (SEs), were extracted and formatted for compatibility with the Joinpoint Regression Program.

Temporal trends were analyzed using Joinpoint regression to estimate annual percent change (APC) and average annual percent change (AAPC) across population subgroups and to assess long-term changes in cervical cancer mortality. To summarize changes over time, the numbers of deaths and AAMR in 1999 and 2023 were compared, with 2020 used for urban–rural stratification because of data availability, and percentage changes were calculated.

Joinpoint regression analyses were performed using the Joinpoint Regression Program (version 4.9.1.0; National Cancer Institute, Bethesda, MD, United States). For the bridged-race female series (1999–2020, 22 annual observations), a maximum of 3 joinpoints was permitted; for the single-race female series (2018–2023, 6 annual observations), a maximum of 1 joinpoint was permitted. The optimal number of joinpoints was determined using the permutation test method with 4,499 randomly permuted datasets and α = 0.05. The Bayesian Information Criterion (BIC) was calculated as a complementary fit measure. The grid search method was applied to identify best-fitting joinpoint locations. The minimum number of observations between consecutive joinpoints and from a joinpoint to either end of the series was set to 2. Autocorrelated errors were assessed using the Durbin-Watson test; when detected, the program’s built-in correction was applied. Annual percent change (APC) and average annual percent change (AAPC) were estimated with 95% confidence intervals using the parametric method; *p* values were derived from the t-distribution. All single-race AAMR values for 2018–2023 were corrected to the ≥25-year female population using the U.S. 2000 standard population weight ratio (×1.54642). The original spliced-series Joinpoint results (prior to the age-restriction correction) are reported in the Supplementary materials for transparency; they are superseded by the corrected analyses presented here.

Spatial analyses were conducted at the state level. Choropleth maps of the United States were generated to visualize the geographic distribution of deaths, AAMR, percentage change in deaths (1999–2023), and AAPC in 2023. Color scales were defined using quantiles or predefined intervals, with blue indicating lower levels and red indicating higher levels, to highlight spatial disparities in cervical cancer mortality burden.

For temporal visualization, line plots were constructed to display annual trends in AAMR across population subgroups. APC values, 95% CIs, and *p* values for each segment were annotated in the figures to facilitate interpretation of the direction and magnitude of temporal changes.

## 3 Results

### 3.1 Overall trends in cervical cancer mortality in the United States, 1999–2023

Overall, the number of cervical cancer–related deaths in the United States remained relatively stable from 1999 to 2023, decreasing slightly from 4,192 to 4,162 deaths (−0.72%), with a temporary increase observed in 2021 (4,366 deaths). In contrast, age-adjusted mortality rate (AAMR) declined from 2.36 per 100,000 (95% CI, 2.29–2.43) in 1999 to 1.09 per 100,000 (95% CI, 1.06–1.12) in 2023, with pronounced reduction occurring during 2019–2023 (AAPC, −3.21%; 95% CI, −4.10 to −2.31) (Table 1; Supplementary Figure S1). The initially observed acceleration in mortality decline after 2019 was not observed after correction of CDC WONDER query conditions. Following adjustment of single-race estimates to the ≥25-year female population, corrected single-race AAMR closely agreed with bridged-race AAMR during overlapping years (2018–2020), with differences within 0.001% (e.g., 2019: 3.30 vs. 3.30 per 100,000). In the bridged-race female series (1999–2020), AAMR declined from 4.4 to 3.4 per 100,000, with Joinpoint regression identifying a single joinpoint in 2004 (APC₁ = −3.5% for 1999–2004; APC₂ = −0.7% for 2004–2020). In the corrected single-race female series (2018–2023), AAMR declined from 3.3 to 3.2 per 100,000 (APC = −0.6%), consistent with the long-term declining trend observed in the bridged-race series. No joinpoint or acceleration in mortality decline was identified around 2019/2020 in either series, indicating that the previously reported post-2019 acceleration reflected differences in data extraction criteria rather than a true change in cervical cancer mortality trends.

**Table 1:** Trends in cervical cancer mortality in the United States, 1999–2023: number of deaths, AAMR, and AAPC.

Demographics	Number of deaths		Percentage change (%)	AAMR		AAPC (95%CI)
	1999	2023		1999	2023	
Both	4192	4162	−0.72	2.36 (2.29 to 2.43)	1.09 (1.06 to 1.12)	−3.21 (−4.10 to −2.31)*
Female	4192	4162	−0.72	4.37 (4.24 to 4.51)	2.09 (2.02 to 2.15)	−3.27 (−3.81 to −2.73)*
Male	0	0				
Northeast	856	585	−31.66	2.39 (2.23 to 2.55)	0.86 (0.78 to 0.93)	−4.23 (−5.31 to −3.13)*
Midwest	905	820	−9.39	2.17 (2.03 to 2.32)	1.02 (0.95 to 1.09)	−3.36 (−3.92 to −2.79)*
South	1670	1932	15.69	2.67 (2.54 to 2.80)	1.31 (1.25 to 1.37)	−3.16 (−3.74 to −2.57)*
West	761	825	8.41	2.04 (1.89 to 2.18)	0.92 (0.86 to 0.98)	−3.54 (−4.47 to −2.59)*
Hispanic	376	649	72.61	2.94 (2.62 to 3.26)	1.20 (1.11 to 1.30)	−4.01 (−5.17 to −2.84)*
NH Black	822	698	−15.09	4.90 (4.56 to 5.25)	1.63 (1.51 to 1.75)	−4.85 (−5.82 to −3.86)*
NH White	2846	2540	−10.75	2.06 (1.98 to 2.14)	1.00 (0.96 to 1.05)	−2.93 (−3.66 to −2.18)*
NH Other	138	269	94.93	2.19 (1.80 to 2.58)	0.85 (0.75 to 0.96)	−4.42 (−5.60 to −3.21)*
Metropolitan^1^	3390	3544	4.54	2.30 (2.22 to 2.38)	1.73 (1.67 to 1.79)	−1.43 (−1.67 to −1.19)*
Nonmetropolitan^1^	802	723	−9.85	2.63 (2.45 to 2.81)	2.09 (1.93 to 2.26)	−1.28 (−1.92 to −0.63)*
25–34 years^2^	217	150	−30.88	0.54 (0.47 to 0.61)	0.33 (0.28 to 0.38)	−2.10 (−4.39 to 0.24)
35–44 years^2^	699	576	−17.60	1.55 (1.44 to 1.67)	1.30 (1.19 to 1.40)	−0.57 (−0.84 to −0.29)*
45–54 years^2^	914	795	−13.02	2.50 (2.34 to 2.66)	1.96 (1.83 to 2.10)	−0.88 (−1.37 to −0.39)*
55–64 years^2^	769	947	23.15	3.23 (3.01 to 3.46)	2.26 (2.12 to 2.41)	−1.46 (−2.02 to −0.90)*
65–74 years^2^	696	885	27.16	3.78 (3.50 to 4.06)	2.55 (2.38 to 2.72)	−1.81 (−2.65 to −0.96)*
75–84 years^2^	568	555	−2.29	4.65 (4.26 to 5.03)	3.02 (2.77 to 3.27)	−2.14 (−2.70 to −1.58)*
85 + years^2^	329	249	−24.32	7.92 (7.06 to 8.78)	4.02 (3.52 to 4.52)	−2.81 (−3.38 to −2.24)*

Statistically significant AAPCs are indicated by an asterisk (*). ^1^For urbanization, the 2023 AAMR was replaced with the 2020 value, and the AAPC was calculated for the period 1999–2020. ^2^ For age groups, AAMR were replaced by crude mortality rates, and AAPCs were calculated based on crude rates. AAMR, Age-Adjusted Mortality Rate; AAPC, Average Annual Percent Change; NH, Non-Hispanic. AAMR estimates for 1999–2020 were derived from the bridged-race female series among women aged ≥25 years. AAMR estimates for 2018–2023 were derived from the single-race female series and harmonized to the ≥25-year female population using a correction factor of 1.54642. Following harmonization, the bridged-race and corrected single-race estimates showed excellent agreement during overlapping years (2018–2020), with differences of less than 0.001%. The “Both” category (based on total-population denominators including males) is provided for comparability with previous reports; however, female-specific AAMR among women aged ≥25 years represents the epidemiologically appropriate measure for cervical cancer mortality assessment. Original spliced-series estimates generated before age harmonization are provided in Supplementary Table S2 for transparency; the present study interpretation is based on the harmonized estimates.

In the initial spliced-series analysis before age harmonization, which combined bridged-race estimates with single-race estimates derived using different age restrictions, the AAMR declined from 1999 to 2019 (APC = −1.21%; 95% CI, −1.67 to −0.74), followed by an apparent steeper decline from 2019 to 2023 (APC = −12.64%; 95% CI, −17.26 to −7.76). This analysis showed an overall reduction in female-specific AAMR from 4.37 to 2.09 per 100,000 women (AAPC = −3.27%; 95% CI, −3.81 to −2.73) (Table 1; Supplementary Figure S1; Supplementary Tables S1, S2). Because the segment-specific estimates were affected by differences in age restrictions between population series, subsequent analyses were based on harmonized estimates using the ≥25-year female population. The initial spliced-series estimates are provided in Supplementary Table S2 for reference.

### 3.2 Racial and ethnic disparities in cervical cancer mortality trends

Overall, non-Hispanic Black women had the highest AAMR throughout the study period, followed by Hispanic women, non-Hispanic White women, and non-Hispanic women of other racial groups.

From 1999 to 2023, the AAMR among non-Hispanic Black women declined markedly from 4.90 to 1.63 per 100,000, representing the largest decrease among all groups (AAPC = −4.85, 95% CI: −5.82 to −3.86), although their rates remained the highest in 2023.

The AAMR in both the non-Hispanic White and non-Hispanic other groups showed overall declining trends. Among non-Hispanic White women, the decline was relatively modest, particularly during 1999–2020 (APC = −0.98, 95% CI: −1.28 to −0.68). After approximately 2013, the mortality curves for non-Hispanic White women and the non-Hispanic other group overlapped, although the AAMR among non-Hispanic White women generally remained higher. A slight increase in AAMR was observed after 2022 in all racial/ethnic groups except non-Hispanic White women (Figure 1; Supplementary Table S3).

**Figure 1:**
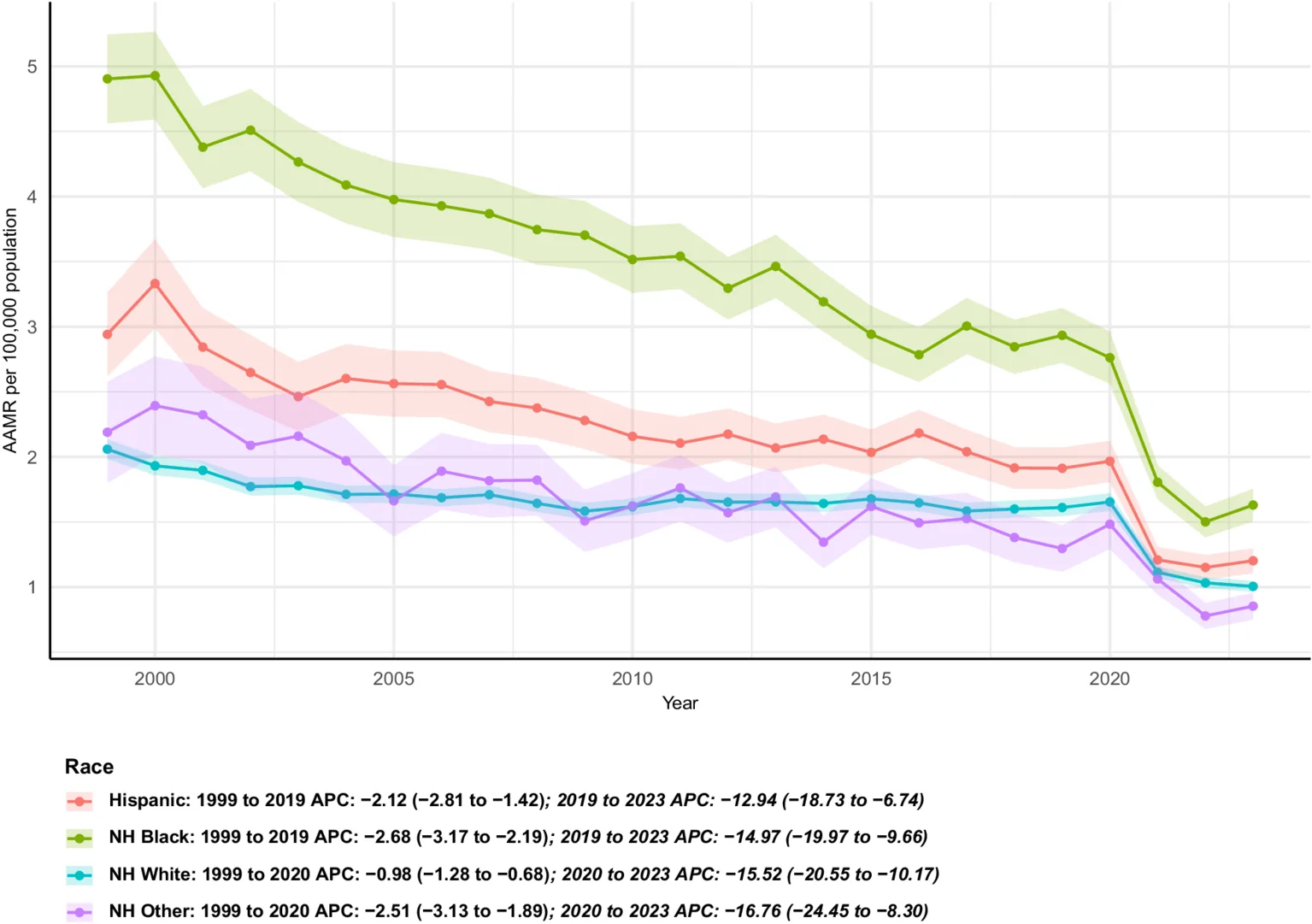
Age-adjusted mortality trends of cervical cancer by race/ethnicity in the United States, 1999–2023. AAMR declined across all racial/ethnic groups. Non-Hispanic Black women had the highest rates throughout the study period despite the largest decline, while non-Hispanic White and non-Hispanic other groups showed overlapping trends after approximately 2013. A slight increase after 2022 was observed in all groups except non-Hispanic White women.

### 3.3 Regional trends and persistent geographic disparities in age-adjusted cervical cancer mortality

Age-adjusted mortality rates (AAMR) declined in all U.S. regions during the study period, with the South consistently showing the highest rates, followed by the Midwest, West, and Northeast. In 2023, the number of cervical cancer–related deaths in the Southern region reached 1,932, corresponding to an AAMR of 1.31 per 100,000 (95% CI: 1.25–1.37). In comparison, the Northeastern region had the lowest rate (0.86 per 100,000). From 1999 to 2019/2020, AAMR decreased significantly across all regions, with the steepest decline observed in the Northeast (APC = −2.22, 95% CI: −2.73 to −1.71), followed by the Midwest, West, and South. Declines in cervical cancer mortality were observed across all four U.S. census regions during the study period, although substantial regional disparities persisted (Figure 2; Supplementary Table S4). In the uncorrected spliced-series analysis shown in Figure 2, the post-2019/2020 segment demonstrated substantially steeper APC estimates, ranging from −13.67 (95% CI: −19.31 to −7.63) in the Northeast to −18.02 (95% CI: −24.19 to −11.34) in the West. However, these accelerated declines were not observed after correction to the ≥25-year female population and were attributable to inconsistent age restrictions during data extraction. Regional Joinpoint analyses based on the corrected estimates confirmed continued declines across all census regions, with persistent differences in the magnitude of decline between regions (Supplementary Table S4).

**Figure 2:**
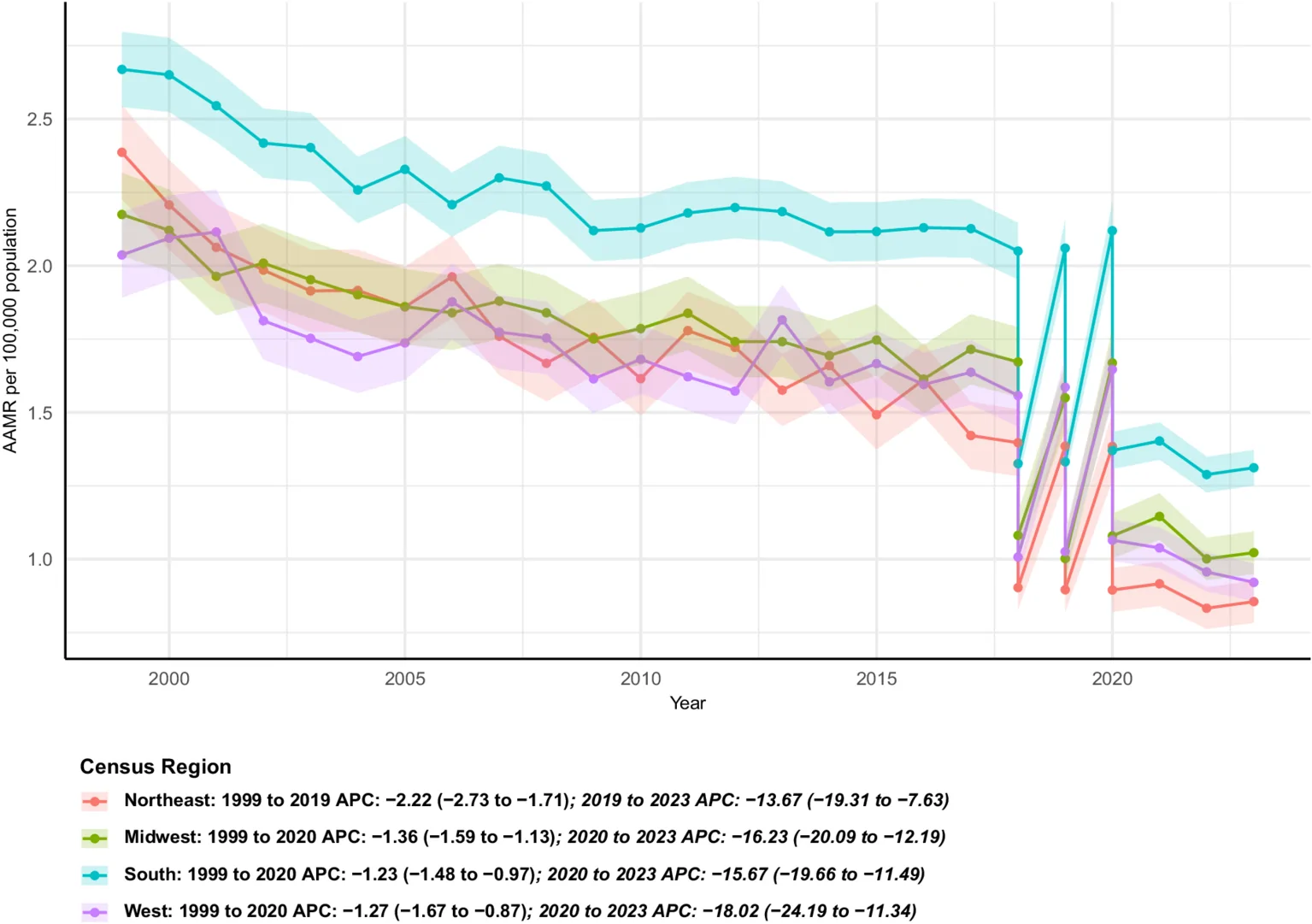
Age-adjusted mortality trends of cervical cancer by census region in the United States, 1999–2023. Age-adjusted mortality trends for cervical cancer are presented by U.S. census region using the original spliced-series estimates. Overall mortality declined across all regions during the study period, with the South consistently showing the highest mortality rates and the Northeast the lowest. The apparent acceleration in mortality decline observed around 2019/2020 should be interpreted cautiously, as it was influenced by differences in CDC WONDER query specifications and age restrictions between population estimation methods. Corrected analyses using harmonized age restrictions and comparable population estimates demonstrated no evidence of a true acceleration in mortality decline.

### 3.4 Trends in AAMR in metropolitan vs. nonmetropolitan areas (1999–2020)

Overall, AAMR declined in both metropolitan and nonmetropolitan areas over the study period, with consistently higher rates in nonmetropolitan areas. In 2020, the AAMR was 2.09 (95% CI: 1.93–2.26) in nonmetropolitan areas and 1.73 (95% CI: 1.67–1.79) in metropolitan areas (Table 1). In metropolitan areas, AAMR decreased significantly from 1999 to 2004 (APC = −3.11, 95% CI: −4.03 to −2.18), followed by a slower but sustained decline from 2004 to 2020. Similarly, in nonmetropolitan areas, AAMR declined markedly during 1999–2004, but the downward trend slowed thereafter, with no statistically significant change from 2004 to 2020 (APC = −0.43, 95% CI: −0.89 to 0.03). Notably, the disparity between metropolitan and nonmetropolitan areas persisted throughout the study period (Figure 3; Supplementary Table S5).

**Figure 3:**
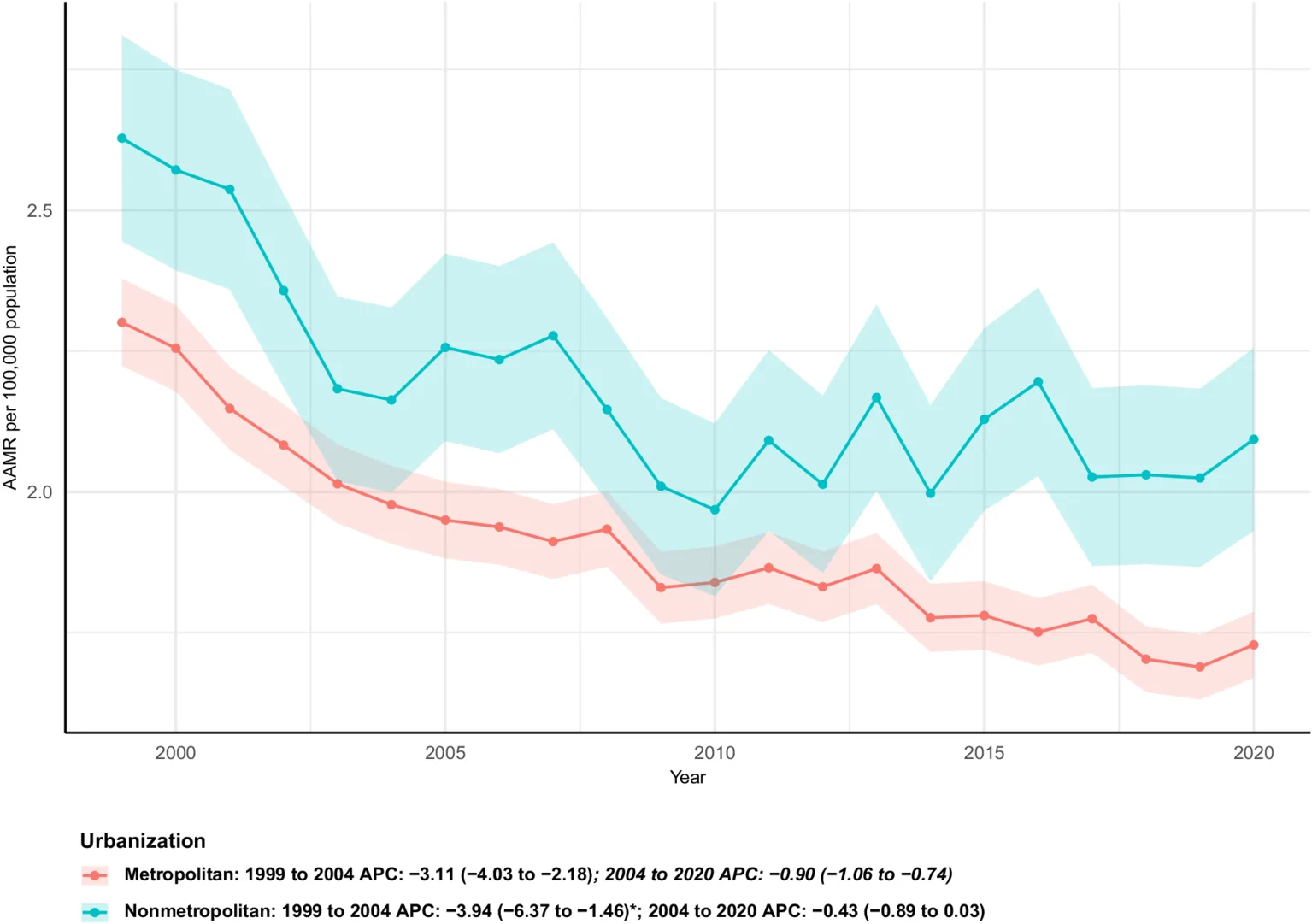
AAMR for cervical cancer in metropolitan and nonmetropolitan areas of the United States, 1999–2020. AAMR declined in both areas over time, but remained consistently higher in nonmetropolitan areas throughout the study period. Shaded areas indicate 95% confidence intervals.

### 3.5 Age-stratified trends in crude mortality rates over time

An age-stratified analysis showed that crude mortality rates declined across all age groups during the study period. The most pronounced decreases were observed among individuals aged ≥85 years and 75–84 years, whereas the smallest changes occurred in the 35–44 and 45–54 year age groups. The AAPC was −0.57 (95% CI: −0.84 to −0.29) in individuals aged 35–44 years, indicating a modest but significant decline, whereas the AAPC in those aged 45–54 years was −0.88 (95% CI: −1.37 to 0.39), indicating a non-significant downward trend. After 2010, age-specific crude mortality rates remained relatively stable across all age groups (Figure 4; Supplementary Table S6).

**Figure 4:**
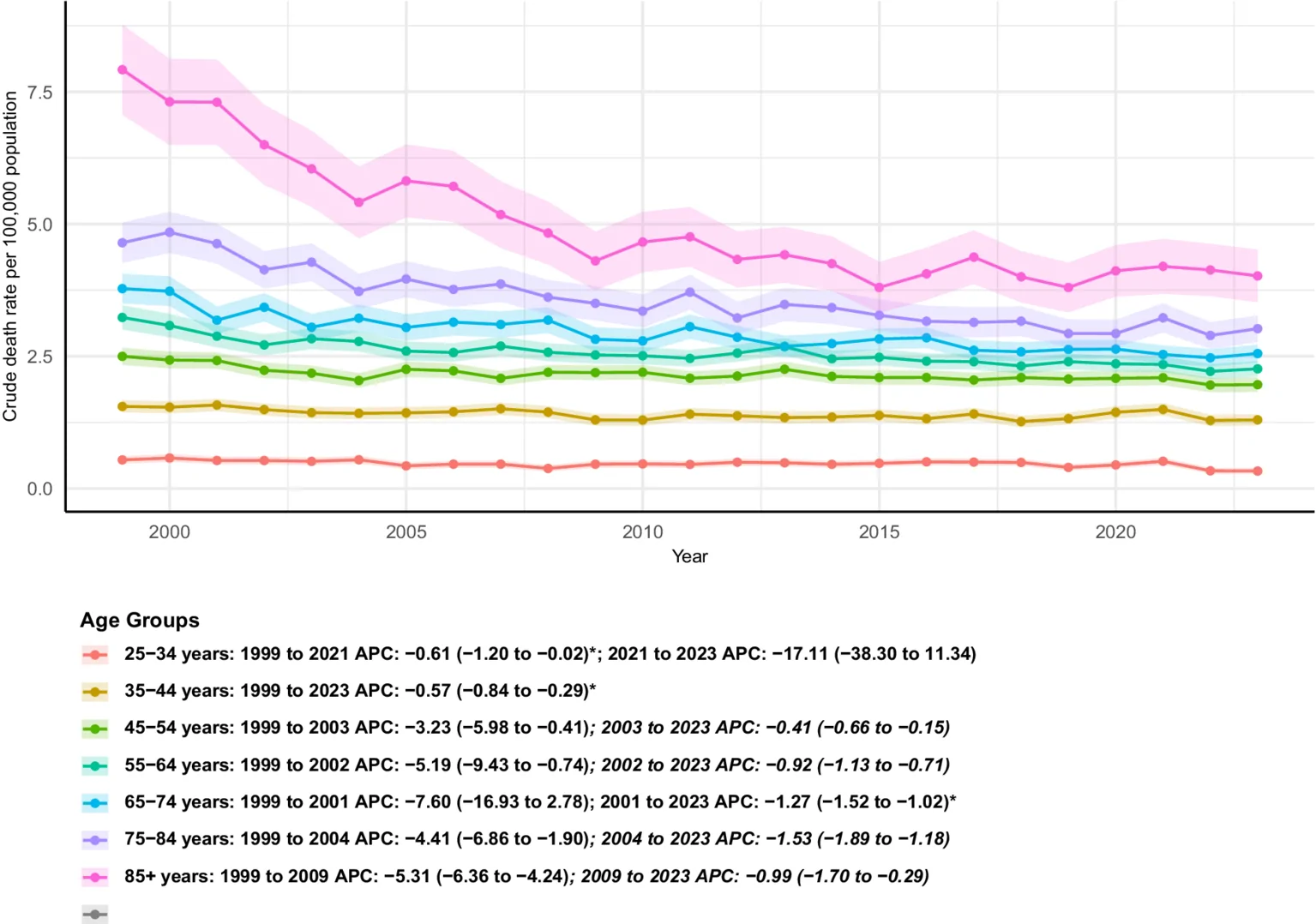
Age-stratified crude mortality rates for cervical cancer in the United States, 1999–2023. Crude mortality rates declined across all age groups over the study period, with the steepest decreases observed among individuals aged ≥85 years and 75–84 years. In contrast, the smallest changes were seen in the 35–44 and 45–54 year age groups. After 2010, age-specific crude mortality rates remained relatively stable across all age groups.

### 3.6 State-level geographic disparities and temporal trends in cervical cancer mortality

Finally, state-level analyses revealed substantial geographic variation in cervical cancer mortality. In 2023, Texas and California had the highest numbers of cervical cancer-related deaths, followed by Florida (Texas: *n* = 453; California: *n* = 450; Florida: *n* = 358) (Figure 5A). Higher AAMR were concentrated in southern states, with the highest rates observed in Mississippi, Arkansas, and Alabama (1.66–1.92 per 100,000). In contrast, the lowest rates were observed in Connecticut (Northeast), as well as Colorado (West) and Minnesota (Midwest) (Figure 5B).

**Figure 5:**
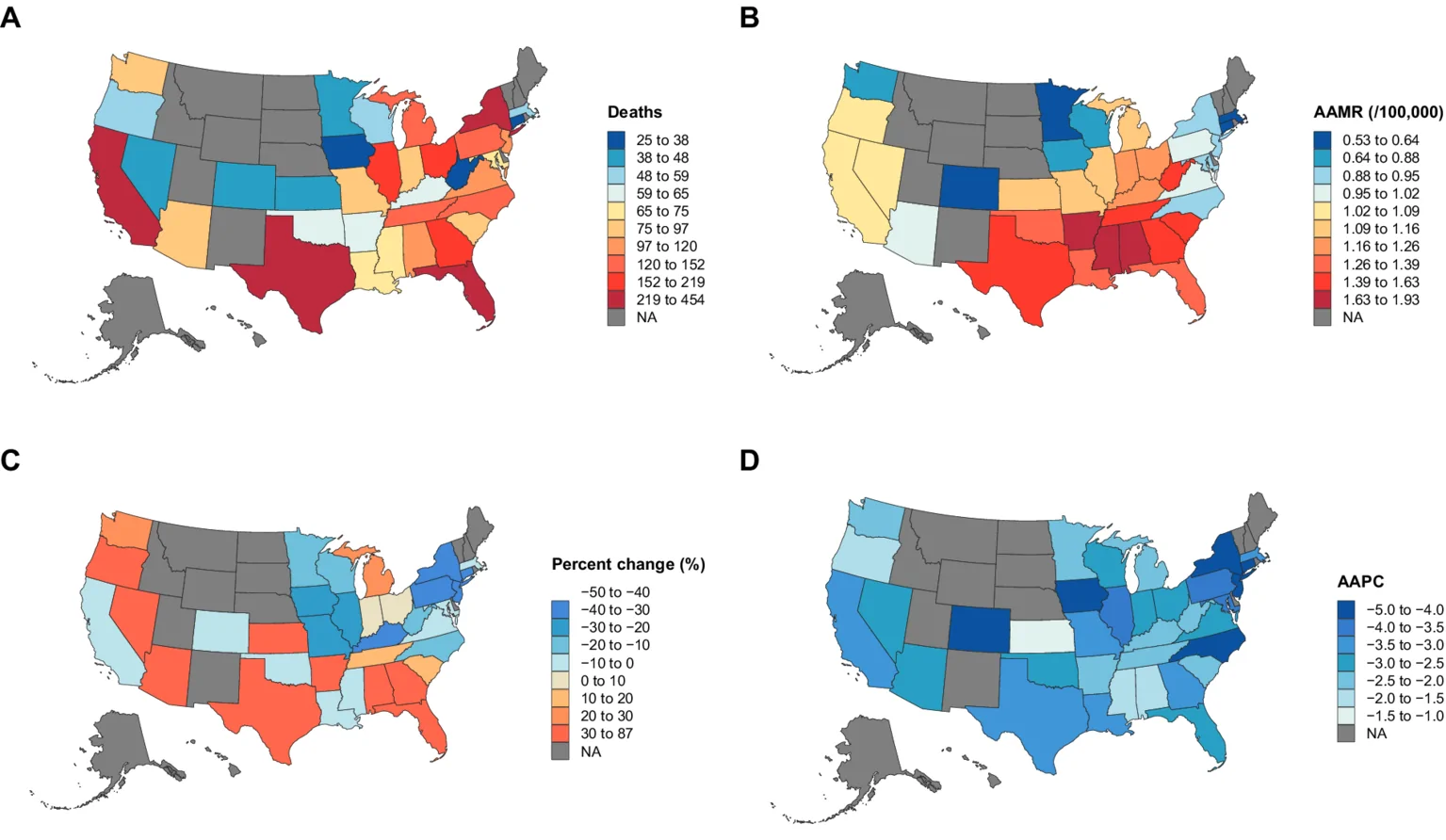
Geographic distribution of cervical cancer mortality across U.S. states in 2023. **(A)** Number of deaths; **(B)** AAMR; **(C)** percentage change in deaths from 1999 to 2023; and **(D)** AAPC. Data were obtained from the CDC WONDER database. AAMR are expressed per 100,000 population and age-standardized to the 2000 U.S. standard population. Color gradients indicate increasing levels of mortality burden, ranging from blue (lowest) to red (highest), based on quantile classification.

From 1999 to 2023, percentage changes in mortality counts varied widely across states, ranging from −50 to +87%. The largest increase was observed in Nevada (West; +86.96%), followed by Alabama (South; +53.97%) and Texas (South; +48.57%), whereas the greatest declines were observed in northeastern states, particularly New York (−35.21%) and New Jersey (−34.78%) (Figure 5C).

State-level AAPCs ranged from −4.0 to −3.0%, indicating overall declines in most states. However, geographic heterogeneity persisted, with the steepest decline observed in New Jersey (Northeast; AAPC: −4.72%; 95% CI: −6.63 to −2.77), followed by Iowa and New York, whereas the smallest declines were observed in Kansas (Midwest; AAPC: −1.0%; 95% CI: −2.30 to 0.31), followed by Oregon (West) and Mississippi (South). Notably, the 95% CI for Kansas crossed zero, indicating that the observed trend was not statistically significant (Figure 5D; Supplementary Table S7). These state-level estimates, particularly for states with small annual death counts, should be interpreted with caution due to potential statistical instability.

## 4 Discussion

This population-based analysis of cervical cancer mortality using CDC WONDER data provides a comprehensive assessment of long-term mortality trends in the United States from 1999 to 2023. After harmonizing age restrictions across population estimation methods, cervical cancer mortality demonstrated a sustained long-term decline, with the most pronounced reduction occurring between 1999 and 2004, followed by a more gradual decline thereafter. Despite this overall improvement, substantial disparities persisted across racial/ethnic, geographic, and urban–rural populations. Non-Hispanic Black women consistently experienced the highest mortality rates, several southern states showed elevated mortality burdens, and women living in nonmetropolitan areas continued to experience higher mortality than those in metropolitan areas. These findings highlight that improvements in cervical cancer mortality have not been equally distributed across populations. Age-specific analyses further indicated relatively limited declines among women aged 35–45 years, highlighting persistent challenges in this age group (Figure 6).

**Figure 6:**
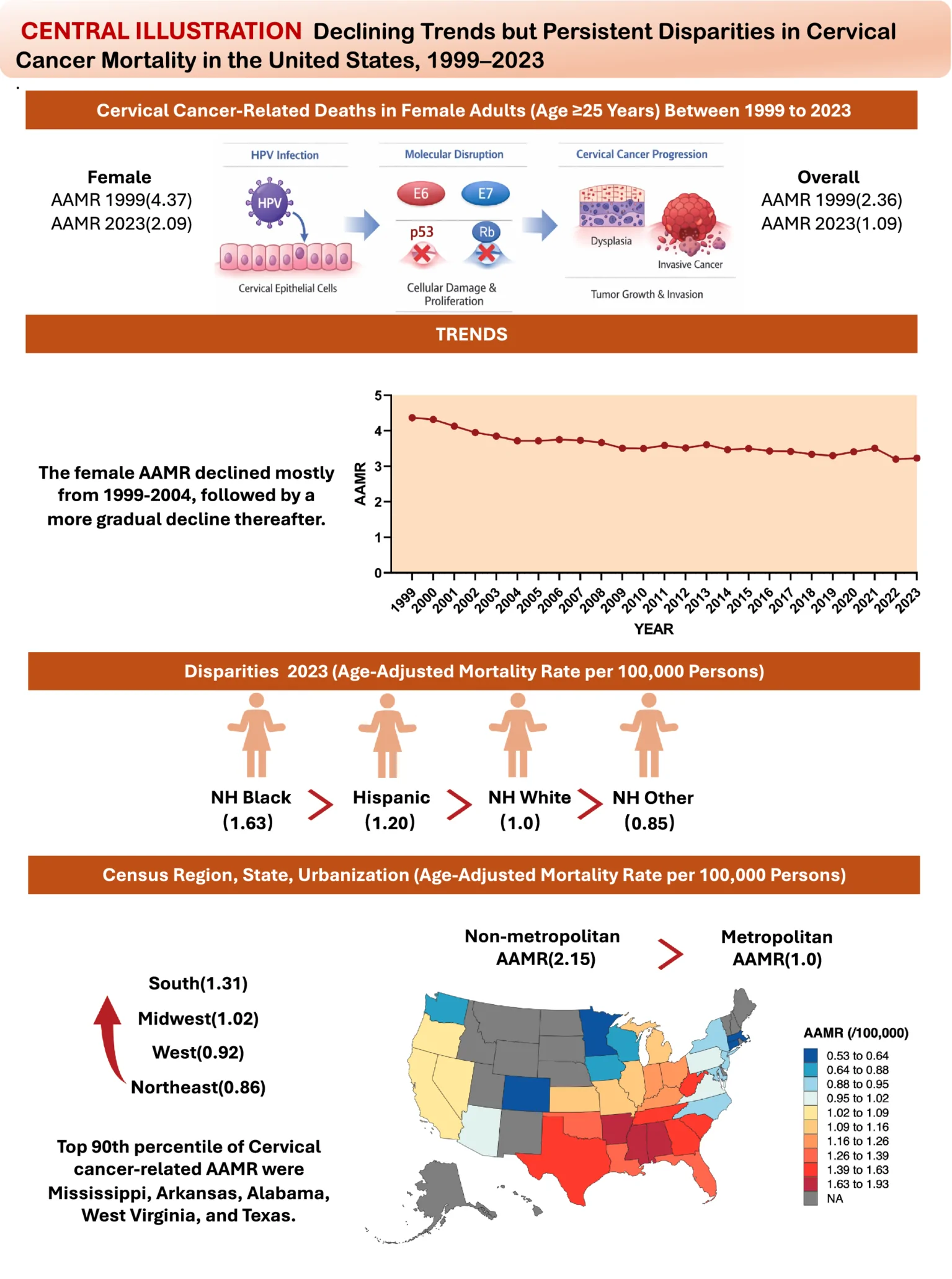
Trends and disparities in cervical cancer mortality in the United States, 1999–2023. Age-adjusted mortality rates (AAMRs) declined overall among U.S. women aged ≥25 years during the study period, with the steepest reductions occurring between 1999 and 2004 and a more gradual decline thereafter. Persistent disparities remained across racial/ethnic groups, geographic regions, states, and urbanization levels. In 2023, non-Hispanic Black women had the highest AAMR (1.63 per 100,000), and mortality rates were highest in the South (1.31 per 100,000) and nonmetropolitan areas (2.15 per 100,000). States with the highest mortality included Mississippi, Arkansas, Alabama, West Virginia, and Texas. Map shading represents AAMRs per 100,000 women, age-standardized to the 2000 U.S. standard population.

A methodological evaluation was performed to investigate the apparent acceleration in cervical cancer mortality decline after 2019/2020. This apparent acceleration was traced to inconsistent age restrictions applied during CDC WONDER data extraction. Specifically, the single-race estimates (2018–2023) were initially extracted without an age restriction, whereas the bridged-race estimates (1999–2020) were restricted to women aged ≥25 years. Because cervical cancer deaths occurred exclusively among women aged ≥25 years in CDC WONDER age-stratified analyses, this discrepancy affected the comparability of the two datasets. After harmonizing age restrictions and population estimation criteria, the single-race and bridged-race estimates showed excellent agreement during overlapping years, indicating that the previously observed post-2019 acceleration reflected methodological inconsistency rather than a true epidemiological change.

Analyses based on internally consistent estimates confirmed the robustness of the long-term declining trend. The bridged-race Joinpoint analysis (1999–2020) identified a single joinpoint in 2004, with a more rapid decline before 2004 and a slower but continued decline thereafter. No joinpoint was identified near 2019/2020 in any population subgroup. Furthermore, the corrected single-race estimates for 2018–2023 showed trends consistent with the long-term decline observed in the bridged-race series. Together, these findings demonstrate that the reduction in cervical cancer mortality represents a sustained secular trend rather than a recent acceleration associated with changes in population estimation methods.

Therefore, the conclusions of this study are based on the corrected analyses presented herein. The original spliced-series estimates are provided in the Supplementary materials for transparency but are not considered primary evidence. This methodological evaluation further emphasizes the importance of harmonizing age restrictions and population estimation criteria when assessing long-term mortality trends using CDC WONDER data.

The rapid initial decline in mortality during the early years of the study period likely reflects the cumulative impact of cervical cancer screening programs that had been widely implemented in the United States by the late twentieth century (14). Pap smear–based screening has long been recognized as one of the most successful cancer prevention strategies, enabling early detection and treatment of precancerous lesions and substantially reducing cervical cancer incidence and mortality (15). National surveillance reports have consistently documented substantial reductions in cervical cancer mortality following the widespread adoption of cytologic screening (16). However, the slower decline observed after the early 2000s may indicate that the benefits of traditional screening programs have approached a plateau, with remaining disease burden increasingly concentrated among populations experiencing limited access to screening and follow-up care (17).

Racial disparities remained among the most striking findings of this analysis. Consistent with previous national studies, non-Hispanic Black women experienced substantially higher cervical cancer mortality than other racial and ethnic groups throughout the study period. Multiple factors likely contribute to this persistent disparity. Evidence suggests that Black women are more likely to be diagnosed with cervical cancer at later stages and may experience delays in receiving guideline-concordant treatment (18). Structural determinants of health—including socioeconomic disadvantage, disparities in insurance coverage, and unequal access to specialized cancer care—may further contribute to these differences (19). These findings reinforce the evidence that racial disparities in cervical cancer outcomes are strongly influenced by inequities in healthcare access and delivery, rather than biological differences alone (20, 21).

After approximately 2013, mortality curves for non-Hispanic White women and non-Hispanic women in other racial groups began to overlap and cross, likely reflecting a slower decline among White women rather than a true increase. Because the “other” racial group is heterogeneous and relatively small, annual age-adjusted rates may be influenced by random variation and potential misclassification of race/ethnicity on death certificates; therefore, these trends should be interpreted with caution (22, 23).

In addition to racial disparities, our findings highlight substantial geographic variation in cervical cancer mortality across the United States. States with relatively higher mortality burdens, including Mississippi, Arkansas, Alabama, West Virginia, and Texas, showed substantially higher mortality rates (approximately two- to four-fold higher) than states with lower burdens, such as Connecticut, Colorado, Minnesota, Massachusetts, and Iowa. These state-level comparisons should be interpreted cautiously, particularly for states with smaller populations or fewer cervical cancer deaths, where estimates may be more sensitive to random variation.

These geographic disparities may reflect structural factors, including differences in screening coverage, socioeconomic conditions, healthcare accessibility, and state-level public health infrastructure (24, 25). Despite overall declines, geographic heterogeneity persisted, suggesting that improvements in cervical cancer prevention and treatment have not occurred uniformly across states.

Urban–rural disparities were also evident in our findings. Women residing in nonmetropolitan areas experienced consistently higher cervical cancer mortality than those living in metropolitan regions, and the rate of decline over time was slower. These results are consistent with a growing body of literature demonstrating worse cancer outcomes among rural populations. Limited availability of gynecologic oncology specialists, longer travel distances to specialized treatment centers, and reduced access to follow-up diagnostic services such as colposcopy may contribute to these differences (26, 27). In addition, rural populations often face socioeconomic barriers affecting healthcare utilization, including transportation limitations, lower insurance coverage, and fewer preventive health services (28, 29). Together, these factors suggest that geographic access to comprehensive cancer care remains an important determinant of cervical cancer outcomes.

Age-specific analysis provided additional insight into mortality trends among women aged 35–45 years. Although mortality declined significantly in this group (APC: −0.57), the magnitude of reduction was modest. Several factors may contribute to this pattern. First, although cervical cancer screening programs target this age group, recent studies have documented declining screening participation among younger women in the United States (30). Second, changes in screening guidelines over the past two decades have extended recommended screening intervals, which may have influenced detection patterns over time (31, 32). Finally, although HPV vaccination was introduced in the United States in 2006 and has demonstrated substantial effectiveness in reducing high-risk HPV infection and cervical precancer, its population-level impact on cervical cancer mortality is expected to become increasingly apparent in future vaccinated cohorts because of the long latency period between HPV infection and invasive cancer development (33).

An additional consideration in interpreting cervical cancer mortality trends is that not all cervical cancers are attributable to HPV infection. Although persistent high-risk HPV infection accounts for the vast majority of cervical cancers, approximately 5–11% of cases may be HPV-negative using conventional detection methods (34). Some of these tumors may represent true HPV-independent malignancies, such as gastric-type endocervical adenocarcinoma, clear cell carcinoma, or mesonephric carcinoma, whereas others may reflect technical limitations or tumor misclassification (35). Because HPV status and histologic subtype were unavailable in the CDC WONDER database, we could not evaluate their contribution to the observed mortality patterns. Nevertheless, these considerations highlight that HPV vaccination and HPV-based screening may not eliminate all cervical cancer risk, emphasizing the continued importance of cervical cancer screening and surveillance.

Compared with previous CDC WONDER-based studies, including Folino et al. (13), our analysis provides several methodological and analytical advances. First, we assessed cervical cancer mortality using female-specific AAMRs among women aged ≥25 years, which more directly reflects mortality burden among the population at risk and avoids dilution caused by inclusion of populations with minimal cervical cancer mortality. Second, we integrated state-level AAMRs with temporal trend analyses using a consistent framework, providing additional insights into geographic heterogeneity and persistent mortality disparities. Given that cancer prevention and screening policies are often implemented at the state level, identifying high-burden areas may help inform targeted interventions and optimize resource allocation.

Several limitations should be considered when interpreting these findings. First, mortality estimates were derived from death certificate data and may be affected by potential misclassification of underlying cause of death, as well as race and ethnicity classification. Second, CDC WONDER does not provide individual-level clinical information, including tumor stage, histologic subtype, screening history, HPV infection status, or treatment details. Therefore, mortality trends alone cannot determine the mechanisms underlying observed changes in cervical cancer mortality. Third, socioeconomic indicators and healthcare access measures are unavailable in CDC WONDER, limiting the ability to account for contextual factors that may influence cervical cancer mortality. Fourth, state-level estimates may be less stable in areas with small populations or few cervical cancer deaths, where rates and temporal trends may be sensitive to random variation.

## 5 Conclusion

This study provides a comprehensive assessment of cervical cancer mortality trends and disparities across demographic and geographic populations in the United States from 1999 to 2023. Although cervical cancer mortality has declined substantially, persistent disparities remain across racial/ethnic groups, geographic regions, and urban–rural populations. After harmonizing age restrictions and population estimation methods, the analyses confirmed a sustained long-term decline in mortality among U.S. women aged ≥25 years, without evidence of a true acceleration after 2019/2020. Continued efforts to expand equitable screening, HPV vaccination, healthcare access, and targeted interventions for high-burden populations are essential to further reduce cervical cancer mortality inequalities.

## Data Availability

The datasets presented in this study can be found in online repositories. The names of the repository/repositories and accession number(s) can be found in the article/Supplementary material.
