# Supplementary material for: Declining trends but persistent disparities in cervical cancer mortality in the United States, 1999–2023
Source: Front Public Health. 2026 Sep 3;14:1892243. doi: 10.3389/fpubh.2026.1892243 (PMC13622166; doi:10.3389/fpubh.2026.1892243)
Supplement: Supplementary file 1 [file Supplementary_file_1.pdf]

Supplemental Table S1 Annual percent change (APC) of cervical cancer–related Age-Adjusted Mortality Rates per 100,000 in Adults in the United States, 1999 to 2023

Year Interval	APC (95% CI)
**Overall**	
1999-2019	−1.21 (−1.67 to −0.74)
2019-2023	-12.64 (-17.26to -7.76)
**Female**	
1999-2020	-1.27(-1.49 to -1.04)
2020-2023	−16.21(−19.90 to −12.35)
**NH White**	
1999-2020	−0.98 (−1.28 to −0.68)
2020-2023	-15.52 (-20.55 to -10.17)
**NH Black**	
1999-2019	−2.68 (−3.17 to −2.19)
2019-2023*	−14.97 (−19.97 to −9.66)
**NH Other**	
1999-2020	−2.51 (−3.13 to −1.89)
2020-2023*	−16.76 (−24.45 to −8.30)
**Hispanic**	
1999-2019	−2.12 (−2.81to −1.42)
2019-2023	-12.94 (-18.73 to -6.74)
**Nonmetropolitan areas**	

1999-2004	−3.11(−4.03 to −2.18)
2004-2020	-0.90 (-1.06 to -0.74)
**Metropolitan area**	
1999-2004	−3.94 (−6.37 to −1.46)
2004-2020	-0.43 (-0.89 to 0.03)

APC = annual percent change; NH = non-Hispanic.

Supplemental Table S2 Overall and Sex-Specific Age-Adjusted Mortality Rates and numbers for Cervical Cancer per 100,000 Adults in the United States, 1999–2023

Year	Women		Overall	
	AAMR	Numbers	AAMR	Numbers
1999	4.37 (4.24-4.51)	4192	2.36（2.29–2.43）	4192
2000	4.32 (4.19-4.46)	4182	2.31（2.24–2.38）	4182
2001	4.13 (4.0-4.25)	4079	2.22（2.15–2.29）	4079
2002	3.95(3.82-4.07)	3935	2.09（2.03–2.16）	3935
2003	3.85(3.73-3.98)	3903	2.04（1.98–2.11）	3903
2004	3.72 (3.81-3.64)	3831	1.96（1.90–2.03）	3831
2005	3.72 (3.81-3.64)	3910	1.99（1.93–2.05）	3910
2006	3.75 (3.63-3.86)	3967	1.98（1.92–2.05）	3967
2007	3.73 (3.62-3.85)	4013	2.00（1.93–2.06）	4013
2008	3.67(3.56-3.79)	3995	1.94（1.88–2.00）	3995
2009	3.51(3.40-3.62)	3888	1.86（1.80–1.92）	3888
2010	3.50 (3.39-3.61)	3927	1.86（1.80–1.92）	3927
2011	3.59 (3.48-3.70)	4079	1.92（1.86–1.98）	4079
2012	3.52(3.41-3.63)	4065	1.87（1.81–1.93）	4065
2013	3.61(3.50-3.72)	4204	1.90（1.84–1.96）	4204
2014	3.47(3.36-3.58)	4103	1.85（1.79–1.90）	4103
2015	3.50 (3.39-3.61)	4165	1.84（1.78–1.90）	4165
2016	3.43 (3.32-3.53)	4180	1.81（1.75–1.87）	4180

2017	3.42 (3.31-3.53)	4199	1.80（1.75–1.86）	4199
2018	3.34(3.23-3.44)	4136	1.76（1.71–1.82）	4136
	2.16(2.09-2.23)*	4138*	1.14（1.1–1.18）*	4138*
2019	3.30 (3.20-3.40)	4144	1.73（1.68–1.78）	4144
	2.13(2.07-2.2) *	4152*	1.12（1.08–1.15）*	4152*
2020	3.41 (3.30-3.52)	4267	1.76（1.71–1.82）	4267
	2.21(2.14-2.27) *	4272*	1.14（1.1–1.17）*	4272*
2021	2.27 (2.20-2.34) *	4366*	1.17（1.13–1.21）*	4366*
2022	2.07 (2.00-2.14) *	4051*	1.07（1.04–1.11）*	4051*
2023	2.09 (2.02-2.15) *	4162*	1.09（1.06–1.12）*	4162*

*Underlying Cause of Death, 2018-2023, Single Race

Supplemental Table S3 Age-Adjusted Cervical Cancer–Related Mortality Rates per 100,000 Adults in the United States, by Race, 1999–2023

Year	NH White	NH Black	NH Other	Hispanic
1999	2.06 (1.98-2.14)	4.90 (4.56–5.25)	2.19 (1.80–2.58)	2.94 (2.62–3.26)
2000	1.93 (1.86-2.00)	4.93 (4.59–5.27)	2.39 (2.02–2.77)	3.33 (2.99–3.67)
2001	1.90 (1.82-1.97)	4.38 (4.06–4.69)	2.32 (1.95–2.69)	2.84 (2.54–3.14)
2002	1.77 (1.70-1.84)	4.51 (4.19–4.83)	2.09 (1.73–2.44)	2.65 (2.36–2.94)
2003	1.78 (1.71-1.85)	4.27 (3.96–4.57)	2.16 (1.81–2.51)	2.46 (2.20–2.73)
2004	1.71 (1.64-1.78)	4.09 (3.80–4.38)	1.97 (1.65–2.29)	2.60 (2.34–2.87)
2005	1.72 (1.65-1.78)	3.98 (3.69–4.26)	1.66 (1.39–1.93)	2.56 (2.31–2.82)
2006	1.69 (1.62-1.75)	3.93 (3.65–4.21)	1.89 (1.59–2.19)	2.56 (2.31–2.81)
2007	1.71 (1.64-1.78)	3.87 (3.59–4.14)	1.82 (1.53–2.10)	2.43 (2.19–2.66)
2008	1.64 (1.58-1.71)	3.75 (3.48–4.01)	1.82 (1.55–2.09)	2.38 (2.15–2.61)
2009	1.58 (1.52-1.65)	3.70 (3.44–3.97)	1.51 (1.27–1.75)	2.28 (2.06–2.50)
2010	1.62 (1.55-1.68)	3.52 (3.26–3.77)	1.62 (1.37–1.87)	2.16 (1.95–2.36)
2011	1.68 (1.61-1.75)	3.54 (3.29–3.79)	1.76 (1.51–2.02)	2.11 (1.90–2.31)
2012	1.65 (1.59-1.72)	3.30 (3.06–3.54)	1.57 (1.34–1.80)	2.17 (1.98–2.37)
2013	1.65 (1.59-1.72)	3.46 (3.22–3.71)	1.69 (1.46–1.92)	2.07 (1.88–2.26)
2014	1.64 (1.58-1.71)	3.19 (2.96–3.42)	1.34 (1.14–1.55)	2.14 (1.95–2.32)
2015	1.68 (1.61-1.74)	2.94 (2.73–3.16)	1.62 (1.40–1.84)	2.03 (1.86–2.21)
2016	1.65 (1.58-1.71)	2.78 (2.58–2.99)	1.49 (1.29–1.70)	2.18 (2.00–2.36)
2017	1.58 (1.52-1.65)	3.01 (2.79–3.22)	1.53 (1.33–1.72)	2.04 (1.87–2.21)

2018	1.60 (1.53-1.66)	2.85（2.64–3.05）	1.38（1.19–1.57）	1.92（1.75–2.08）
2019	1.61 (1.54-1.68)	2.93（2.72–3.14）	1.30（1.12–1.47）	1.91（1.75–2.07）
2020	1.65 (1.59-1.72)	2.76（2.56–2.96）	1.48（1.29–1.67）	1.97（1.81–2.12）
2021	1.11 (1.07-1.16)	1.81（1.67–1.94）	1.06（0.94–1.88）	1.21（1.11–1.31）
2022	1.03(0.99-1.07)	1.50（1.38–1.62）	0.78（0.68–0.88）	1.15（1.05–1.25）
2023	1.00(0.96-1.05)	1.63（1.51–1.75）	0.85（0.75–0.96）	1.20（1.11–1.30）

NH = non-Hispanic.

Supplemental Table S4 Age-Adjusted Cervical Cancer–Related Mortality Rates per 100,000 Adults in the United States, by Census Region, 1999–2023

Year	Northeast	Midwest	South	West
1999	2.39 (2.23–2.55)	2.17 (2.03–2.32)	2.67 (2.54–2.80)	2.04 (1.89–2.18)
2000	2.21 (2.05–2.36)	2.12 (1.98–2.26)	2.65 (2.52–2.78)	2.09 (1.95–2.24)
2001	2.06 (1.92–2.21)	1.96 (1.83–2.10)	2.55 (2.42–2.67)	2.11 (1.97–2.26)
2002	1.99 (1.84–2.13)	2.01 (1.87–2.14)	2.42 (2.30–2.54)	1.81 (1.68–1.95)
2003	1.91 (1.77–2.05)	1.95 (1.82–2.08)	2.40 (2.29–2.52)	1.75 (1.62–1.88)
2004	1.92 (1.78–2.06)	1.90 (1.77–2.03)	2.26 (2.15–2.37)	1.69 (1.57–1.81)
2005	1.86 (1.72–2.00)	1.86 (1.73–1.99)	2.33 (2.22–2.44)	1.74 (1.61–1.86)
2006	1.96 (1.82–2.10)	1.84 (1.71–1.97)	2.21 (2.10–2.32)	1.88 (1.75–2.01)
2007	1.76 (1.63–1.89)	1.88 (1.75–2.01)	2.30 (2.19–2.41)	1.77 (1.65–1.90)
2008	1.67 (1.54–1.80)	1.84 (1.71–1.96)	2.27 (2.16–2.38)	1.75 (1.63–1.88)
2009	1.76 (1.62–1.89)	1.75 (1.63–1.87)	2.12 (2.02–2.22)	1.61 (1.50–1.73)
2010	1.61 (1.49–1.74)	1.79 (1.66–1.91)	2.13 (2.02–2.23)	1.68 (1.56–1.80)
2011	1.78 (1.65–1.91)	1.84 (1.71–1.96)	2.18 (2.07–2.28)	1.62 (1.51–1.74)
2012	1.72 (1.59–1.85)	1.74 (1.62–1.86)	2.20 (2.09–2.30)	1.57 (1.46–1.69)
2013	1.58 (1.45–1.70)	1.74 (1.62–1.86)	2.18 (2.08–2.29)	1.82 (1.69–1.94)
2014	1.66 (1.53–1.79)	1.69 (1.57–1.81)	2.12 (2.01–2.22)	1.60 (1.49–1.72)
2015	1.49 (1.37–1.61)	1.75 (1.63–1.87)	2.12 (2.02–2.22)	1.67 (1.55–1.78)
2016	1.61 (1.49–1.74)	1.61 (1.50–1.73)	2.13 (2.03–2.23)	1.59 (1.49–1.70)
2017	1.42 (1.31–1.54)	1.72 (1.60–1.83)	2.13 (2.03–2.23)	1.64 (1.53–1.75)

2018	1.40（1.28–1.51）<br>0.90(0.83–0.98) *	1.67（1.55–1.79）<br>1.08（1.0–1.16）*	2.05（1.95–2.15）<br>1.33(1.26–1.39) *	1.56（1.45–1.66）<br>1.0（0.94–1.08）*
2019	1.39（1.27–1.50）<br>0.90（0.82–0.97）*	1.55（1.44–1.66）<br>1.0（0.93–1.07）*	2.06（1.96–2.16）<br>1.33(1.27–1.390*	1.59（1.48–1.69）<br>1.02（0.96–1.09）*
2020	1.38（1.27–1.50）<br>0.89（0.82–0.97）*	1.67（1.55–1.79）<br>1.08（1.0–1.16）*	2.12（2.02–2.22）<br>1.37(1.30–1.43)*	1.65（1.54–1.76）<br>1.06（1.00–1.14）*
2021	0.92 (0.84-0.99) *	1.15(1.07–1.22) *	1.40(1.34–1.47) *	1.04 (0.97–1.11) *
2022	0.83(0.76-0.90) *	1.00(0.93–1.07) *	1.29(1.23–1.35) *	0.96 (0.89–1.02) *
2023	0.86(0.78-0.93) *	1.02(0.95–1.09) *	1.31(1.25–1.37) *	0.92 (0.86–0.98) *

*Underlying Cause of Death, 2018-2023, Single Race

Supplemental Table S5 Age-Adjusted Cervical Cancer–Related Mortality Rates per 100,000 Adults in the United States, by Urban–Rural Classification, 1999–2020

	**Age-Adjusted Rate (95% CI)**	
**Year**	**Metropolitan**	**Nonmetropolitan**
1999	2.30（2.22–2.38）	2.63（2.45–2.81）
2000	2.25（2.18–2.33）	2.57（2.39–2.75）
2001	2.15（2.07–2.22）	2.54（2.36–2.71）
2002	2.08（2.01–2.16）	2.36（2.19–2.53）
2003	2.01（1.94–2.08）	2.18（2.02–2.35）
2004	1.98（1.91–2.05）	2.16（2.00–2.33）
2005	1.95（1.88–2.02）	2.26（2.09–2.42）
2006	1.94（1.87–2.00）	2.23（2.07–2.40）
2007	1.91（1.85–1.98）	2.28（2.11–2.44）
2008	1.93（1.87–2.00）	2.15（1.98–2.31）
2009	1.83（1.77–1.89）	2.01（1.85–2.17）
2010	1.84（1.78–1.90）	1.97（1.81–2.12）
2011	1.87（1.80–1.93）	2.09（1.93–2.25）
2012	1.83（1.77–1.89）	2.01（1.86–2.17）
2013	1.86（1.80–1.93）	2.17（2.00–2.33）
2014	1.78（1.72–1.84）	2.00（1.84–2.15）
2015	1.78（1.72–1.84）	2.13（1.97–2.29）

2016	1.75（1.69–1.81）	2.20（2.03–2.36）
2017	1.77（1.71–1.84）	2.03（1.87–2.18）
2018	1.70（1.64–1.76）	2.03（1.87–2.19）
2019	1.69（1.63–1.75）	2.02（1.87–2.18）
2020	1.73（1.67–1.79）	2.09（1.93–2.26）

Supplementary Table S6 Trends in Cervical Cancer Mortality by Age Group in the United States, 1999–2023

Year	25-34 years	35-44 years	45-54 years	55-64 years	65-74 years	75-84 years	85+ years
1999	0.54 (0.47–0.61)	1.55 (1.44–1.67)	2.50 (2.34–2.66)	3.23 (3.01–3.46)	3.78 (3.50–4.06)	4.65 (4.26–5.03)	7.92 (7.06–8.78)
2000	0.58 (0.50–0.65)	1.54 (1.42–1.65)	2.43 (2.27–2.59)	3.08 (2.86–3.30)	3.73 (3.45–4.01)	4.85 (4.46–5.23)	7.31 (6.50–8.13)
2001	0.53 (0.46–0.60)	1.58 (1.46–1.69)	2.42 (2.27–2.57)	2.88 (2.67–3.09)	3.18 (2.92–3.44)	4.63 (4.25–5.01)	7.30 (6.50–8.11)
2002	0.53 (0.46–0.60)	1.49 (1.38–1.61)	2.24 (2.09–2.38)	2.72 (2.52–2.91)	3.43 (3.16–3.69)	4.14 (3.78–4.49)	6.50 (5.74–7.26)
2003	0.51 (0.44–0.58)	1.43 (1.32–1.55)	2.18 (2.04–2.32)	2.83 (2.63–3.03)	3.05 (2.80–3.30)	4.28 (3.92–4.64)	6.05 (5.32–6.77)
2004	0.54 (0.47–0.62)	1.42 (1.31–1.53)	2.04 (1.90–2.18)	2.78 (2.59–2.97)	3.22 (2.96–3.48)	3.73 (3.39–4.06)	5.41 (4.74–6.09)
2005	0.43 (0.36–0.49)	1.43 (1.32–1.54)	2.25 (2.11–2.40)	2.60 (2.42–2.78)	3.05 (2.80–3.29)	3.96 (3.62–4.30)	5.82 (5.13–6.51)
2006	0.46 (0.39–0.53)	1.45 (1.34–1.56)	2.22 (2.08–2.37)	2.57 (2.40–2.75)	3.15 (2.89–3.40)	3.76 (3.43–4.10)	5.71 (5.04–6.38)
2007	0.46 (0.39–0.53)	1.51 (1.39–1.62)	2.08 (1.95–2.22)	2.69 (2.52–2.87)	3.10 (2.86–3.35)	3.87 (3.53–4.20)	5.18 (4.55–5.81)
2008	0.38 (0.32–0.44)	1.44 (1.33–1.56)	2.20 (2.06–2.34)	2.58 (2.41–2.75)	3.18 (2.94–3.43)	3.62 (3.29–3.94)	4.83 (4.23–5.43)

2009	0.46（0.39–0.53）	1.30（1.19–1.41）	2.19（2.05–2.33）	2.53（2.36–2.69）	2.82（2.60–3.05）	3.50（3.18–3.82）	4.30（3.75–4.86）
2010	0.47（0.40–0.53）	1.29（1.18–1.40）	2.20（2.06–2.33）	2.51（2.35–2.67）	2.79（2.57–3.01）	3.35（3.04–3.67）	4.66（4.09–5.23）
2011	0.45（0.39–0.52）	1.41（1.29–1.52）	2.08（1.95–2.22）	2.46（2.30–2.62）	3.06（2.83–3.29）	3.71（3.38–4.04）	4.76（4.19–5.32）
2012	0.50（0.43–0.56）	1.37（1.26–1.49）	2.13（1.99–2.26）	2.56（2.40–2.72）	2.86（2.65–3.07）	3.22（2.92–3.53）	4.33（3.80–4.86）
2013	0.49（0.42–0.55）	1.34（1.23–1.45）	2.26（2.11–2.40）	2.68（2.52–2.84）	2.69（2.49–2.89）	3.48（3.17–3.80）	4.42（3.89–4.95）
2014	0.46（0.39–0.52）	1.35（1.24–1.46）	2.12（1.98–2.26）	2.45（2.30–2.61）	2.74（2.54–2.94）	3.42（3.11–3.73）	4.25（3.74–4.77）
2015	0.48（0.41–0.54）	1.38（1.27–1.50）	2.10（1.96–2.23）	2.48（2.33–2.63）	2.83（2.63–3.03）	3.28（2.97–3.58）	3.80（3.32–4.28）
2016	0.50（0.44–0.57）	1.32（1.21–1.43）	2.10（1.96–2.24）	2.41（2.26–2.56）	2.85（2.65–3.05）	3.16（2.87–3.45）	4.06（3.56–4.55）
2017	0.50（0.43–0.56）	1.41（1.29–1.52）	2.05（1.91–2.19）	2.40（2.25–2.55）	2.61（2.43–2.80）	3.14（2.85–3.43）	4.37（3.87–4.88）
2018	0.49（0.43–0.56）	1.26（1.16–1.37）	2.10（1.96–2.24）	2.32（2.17–2.46）	2.58（2.40–2.76）	3.16（2.88–3.44）	4.00（3.52–4.49）
2019	0.40（0.34–0.46）	1.32（1.21–1.43）	2.07（1.93–2.21）	2.40（2.25–2.55）	2.63（2.45–2.81）	2.93（2.67–3.20）	3.80（3.33–4.27）
2020	0.44（0.38–0.51）	1.44（1.33–1.56）	2.08（1.94–2.22）	2.36（2.21–2.50）	2.64（2.46–2.81）	2.93（2.67–3.19）	4.12（3.63–4.60）

2021	0.51(0.45-0.58)	1.50（1.38–1.61）	2.09（1.95–2.23）	2.35（2.20–2.49）	2.53（2.36–2.70）	3.23（2.95–3.5）	4.20（3.68–4.72）
2022	0.33(0.28-0.39)	1.29（1.18–1.39）	1.96（1.82–2.09）	2.21（2.07–2.36）	2.47（2.30–2.64）	2.89（2.64–3.15）	4.13（3.64–4.63）
2023	0.33(0.28-0.38)	1.30（1.19–1.40）	1.96（1.83–2.10）	2.26（2.12–2.41）	2.55（2.38–2.72）	3.02（2.77–3.27）	4.02（3.52–4.52）

Supplemental Table S7 Geographic Distribution of Cervical Cancer in the United States: Number of Deaths, Age-Adjusted Mortality Rates (AAMR) in 2023, Percent Change, and Average Annual Percent Change (AAPC) by State, 1999–2023

	Deaths 2023	AAMR (95% CI) 2023	Percent Change（%） 1999-2023	AAPC 1999-2023
Alabama	97	1.66 (1.33-2.04)	53.97	-1.91 (-3.67--0.12)
Alaska	10	NA	NA	NA
Arizona	80	1.01 (0.79-1.27)	45.45	-2.97 (-4.81--1.10)
Arkansas	64	1.80 (1.37-2.32)	39.13	-2.24 (-3.45--1.01)
California	450	1.05 (0.95-1.15)	-1.53	-3.39 (-4.76--2.01)
Colorado	40	0.60 (0.43-0.82)	-2.44	-4.17 (-7.07--1.17)
Connecticut	26	0.54 (0.35-0.81)	-31.58	-4.37 (-7.66--0.96)
Delaware	11	NA	-47.62	NA
Florida	358	1.26 (1.12-1.40)	32.10	-2.99 (-4.17--1.79)
Georgia	165	1.39 (1.17-1.61)	32.00	-3.03 (-4.49--1.55)
Idaho	14	NA	27.27	NA
Illinois	160	1.09 (0.92-1.27)	-26.61	-3.90 (-5.06--2.72)
Indiana	88	1.16 (0.93-1.44)	3.53	-2.60 (-4.28--0.89)
Iowa	31	0.80 (0.53-1.14)	-24.39	-4.46 (-6.69--2.16)
Kansas	38	1.14 (0.79-1.58)	40.74	-1.00 (-2.30-0.31)
Kentucky	59	1.18 (0.89-1.54)	-32.95	-2.01 (-3.01--0.99)

Louisiana	65	1.28 (0.98-1.64)	-4.41	-3.38 (-5.27--1.46)
Maine	16	NA	-15.79	NA
Maryland	69	0.91 (0.70-1.15)	-9.21	-3.69 (-5.23--2.13)
Massachusetts	57	0.64 (0.48-0.83)	-9.52	-3.20 (-4.09--2.30)
Michigan	129	1.10 (0.90-1.30)	20.56	-2.34 (-3.69--0.96)
Minnesota	42	0.63 (0.45-0.86)	-12.50	-2.38 (-3.25--1.50)
Mississippi	65	1.92 (1.47-2.47)	-5.80	-1.80 (-2.62--0.96)
Missouri	75	1.11 (0.87-1.41)	-25.00	-3.46 (-5.27--1.60)
Nebraska	18	NA	-28.00	NA
Nevada	43	1.09 (0.78-1.47)	86.96	-2.78 (-5.14--0.37)
New Hampshire	10	NA	-23.08	NA
New Jersey	99	0.90 (0.72-1.10)	-34.87	-4.72 (-6.63--2.77)
New Mexico	22	0.90 (0.55-1.39)	0	NA
New York	219	0.91 (0.78-1.03)	-35.21	-4.44 (-5.66--3.20)
North Carolina	120	0.95 (0.77-1.12)	-14.29	-4.04 (-5.60--2.46)
Ohio	168	1.23 (1.03-1.42)	0.60	-2.97 (-4.56--1.35)
Oklahoma	63	1.36 (1.04-1.76)	-3.08	-2.78 (-5.87-0.41)
Oregon	52	1.04 (0.77-1.38)	48.57	-1.69 (-2.96--0.41)
Pennsylvania	145	0.96 (0.80-1.12)	-32.24	-3.66 (-5.15--2.15)
South Carolina	84	1.39 (1.10-1.74)	15.07	-2.27 (-2.93--1.60)
South Dakota	11	NA	NA	NA

Tennessee	124	1.49 (1.22-1.76)	15.89	-2.32 (-3.26--1.38)
Texas	453	1.49 (1.35-1.62)	50.00	-3.11 (-4.21--2.01)
Utah	17	NA	13.33	NA
Virginia	98	0.96 (0.78-1.18)	-1.01	-2.72 (-3.63--1.80)
Washington	75	0.86 (0.67-1.08)	27.12	-2.01 (-3.11--0.89)
West Virginia	33	1.63 (1.09-2.34)	-15.38	-2.14 (-3.23--1.04)
Wisconsin	57	0.81 (0.61-1.06)	-17.39	-2.62 (-3.67--1.55)
