# Supplementary material for: Declining trends but persistent disparities in cervical cancer mortality in the United States, 1999–2023
Source: Front Public Health. 2026 Sep 3;14:1892243. doi: 10.3389/fpubh.2026.1892243 (PMC13622166; doi:10.3389/fpubh.2026.1892243)
Supplement: Supplementary file 2 [file Supplementary_file_2.pdf]

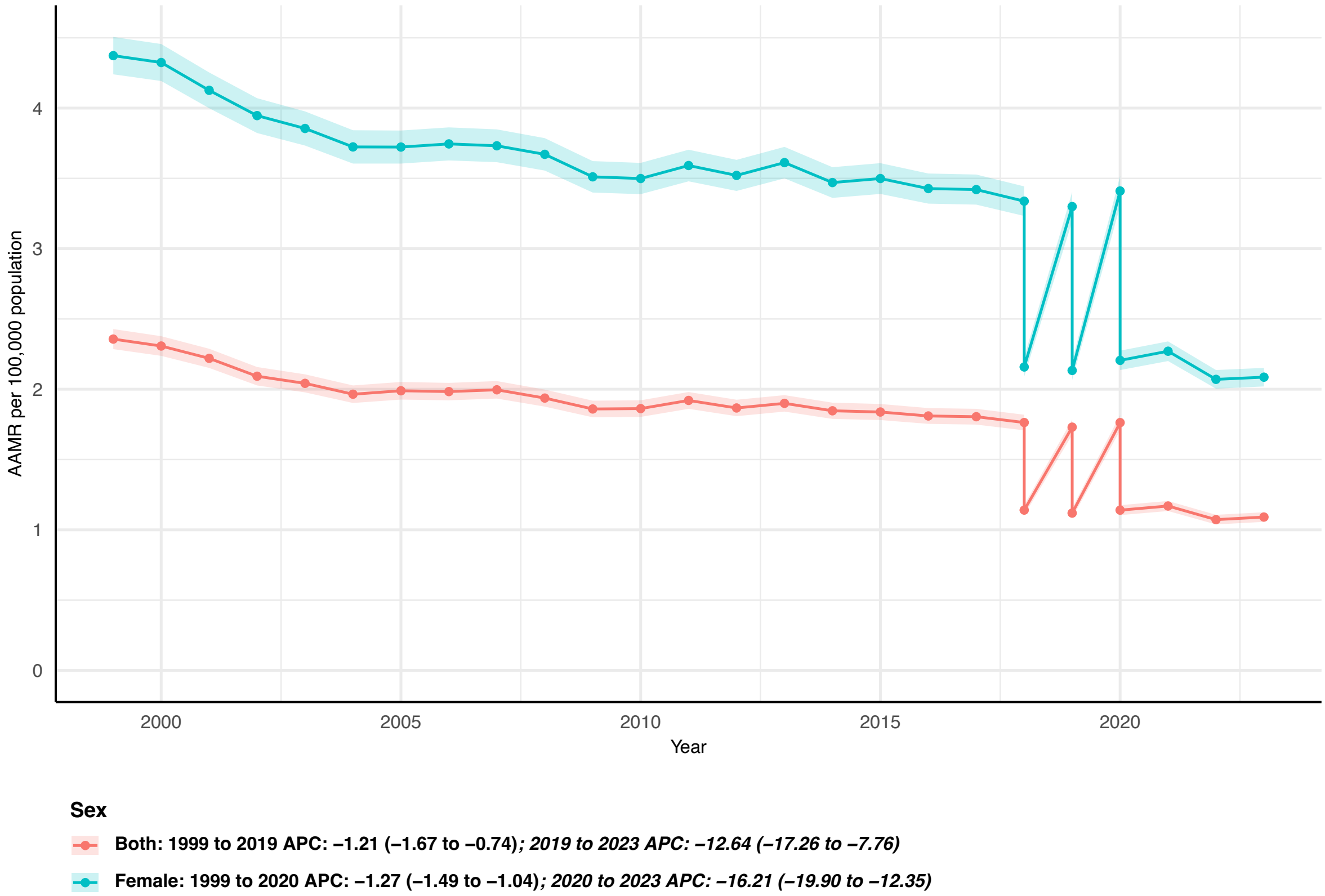

Figure S1. Age-adjusted mortality trends of Cervical Cancer by sex in the United States, 1999–2023.Age-adjusted mortality trends for cervical cancer are presented by U.S. census region using the original spliced-series estimates. Overall mortality declined during the study period. The apparent acceleration in mortality decline observed around 2019/2020 should be interpreted cautiously, as it was influenced by differences in CDC WONDER query specifications and age restrictions between population estimation methods.
